# DOA Estimation for Coherent Sources Based on Uniformly Distributed Two Concentric Rings Array

**DOI:** 10.3390/s23208408

**Published:** 2023-10-12

**Authors:** Chuang Han, Shenghong Guo, Ning Yan, Jingwei Dong, Bowen Xing

**Affiliations:** 1 Heilongjiang Province Key Laboratory of Laser Spectroscopy Technology and Application, Harbin University of Science and Technology, Harbin 150080, China; hanchuang501@163.com (C.H.); 1705030305@stu.hrbust.edu.cn (S.G.); yanning@mskj.com (N.Y.); 2 College of Engineering Science and Technology, Shanghai Ocean University, Shanghai 201306, China

**Keywords:** coherent sources, DOA, concentric rings array

## Abstract

The direction estimation of the coherent source in a uniform circular array is an essential part of the signal processing area of the array, but the traditional uniform circular array algorithm has a low localization accuracy and a poor localization effect on the coherent source. To solve this problem, this paper proposes a two-dimensional direction of arrival (DOA) estimation for the coherent source in broadband. Firstly, the central frequency of the coherent sound source is estimated using the frequency estimation method of the delayed data, and a real-valued beamformer is constructed using the concept of the multiloop phase mode. Then, the cost function in the beam space is obtained. Finally, the cost function is searched in two dimensions to locate the sound source. In this paper, we simulate the DOA of the sound source at different frequencies and signal-to-noise ratios and analyze the resolution of the circular array. The simulation results show that the proposed algorithm can estimate the direction of arrival with high precision and achieve the desired results.

## 1. Introduction

The accurate tracking and DOA of enemy underwater vehicles are an effective means to ensure their security and reduce international disputes. When an underwater enemy vehicle is tracked, it often adopts evasive means of transmitting acoustic decoys [1,2] and self-silence. During this process, it is possible to track both the enemy body and the acoustic decoy for a short period of time, and then the enemy equipment body will quickly go silent or even emit bubble curtains to hide its radiation noise. Therefore, it is imperative to take advantage of this short moment to locate the underwater vehicle and the acoustic decoy of the other party. It is also an essential means of distinguishing between the acoustic decoy and the body of the enemy equipment. The acoustic decoy is almost identical to the radiation noise of the equipment itself, so the acoustic decoy and the equipment itself can be regarded as coherent sound sources. In general underwater acoustic signal processing, the similarity of two time-domain signals is referred to as “correlation”, and the similarity of their frequency domain is called “coherence”. Two signal sources with a coherence exceeding 0.8 are commonly termed as “strongly coherent sources” or simply “coherent sources”. If two signal sources are coherent sources, their relationship can be represented using a linear equation [3,4,5]. Therefore, DOA and identification of coherent sound sources are essential research directions. The passive detection of the direction of arrival of spatial signals is a conventional detection method for underwater ships, and the uniform linear array (ULA) is usually used to estimate the one-dimensional DOA. In practical applications, 360-degree scanning and 180-degree scanning are required for the horizontal and vertical directions of the signal source, whereas the gain and directivity of the linear array vary with the range of scanning angle, which limits the practical application of the linear array to some extent [6,7,8,9,10]. In comparison, the uniform circular array has an excellent omnidirectional scanning ability in both horizontal and vertical directions, and, in general, the range of direction-finding of the uniform circular array is larger than that of the uniform linear array [11]. Especially for large underwater equipment, the topology structure of the array often directly affects the DOA results because of spatial limitations. It is precisely because of such advantages of the uniform circular array that the two-dimensional DOA estimation based on the uniform circular array has been developed rapidly at present.

Conventional DOA algorithms for sound sources are based on the incoherent source. Multiple signal classification (MUSIC) [12,13,14] proposed by Schmidt in 1979 and estimation signal parameter via rotational invariance techniques (ESPRIT) proposed by Roy et al. in 1986 have realized the leap from traditional direction finding to modern high-resolution direction finding, opening up a new field of feature subspace classification algorithms [15,16,17,18,19]. In the late 1980s, weighted subspace fitting (WSF) was proposed to transform such problems into optimization problems with multidimensional parameters [20,21,22,23,24]. The spatial smoothing algorithm is one of the effective means to solve the DOA of the coherent sound source, but it is applied mainly to the linear array, and the effect of the circular array is not ideal [25,26,27]. The utilization of orbital angular momentum antennas for target localization represents an advanced technology. By effectively leveraging the antenna’s orbital angular momentum to achieve target localization, precision and efficiency in positioning can be significantly improved. This technique has a wide range of applications across various domains, including radar systems, communication systems, and navigation systems, providing robust support for precise target positioning and tracking. Additionally, the synthesis of low-sidelobe vortex waves holds promise to provide a theoretical foundation for the future development of secure vortex communication and vortex radar technologies [28,29].

It is well known that increasing the number of array sensors is an effective means to improve the DOA performance of the array when the aperture of the receiving array is fixed, but increasing the number of sensors in a single ring is not an effective means. If these sensors are uniformly arranged on a circular surface, they can be used as a plane array to estimate the DOA of the sound sources. However, in practical engineering applications, because there is mutual occlusion between the sensors, which affects the reception of the signal, the placement of the sensors is not as dense as possible. Therefore, it is necessary to control the distance between sensors while increasing the number of sensors, and at the same time ensure that the position of sensors has certain rules, which is convenient to establish the array manifold matrix.

In this paper, we propose a method to estimate the two-dimensional DOA of the coherent broadband source by using the two concentric rings array based on existing algorithms. Through the simulation analysis, under the premise that the number of required array elements corresponds to the size of the underwater platform, the two-dimensional DOA estimation of the broadband coherent sound source has a higher estimation accuracy and shows a better positioning effect under the condition of different SNRs and different central frequencies.

**Figure 1:**
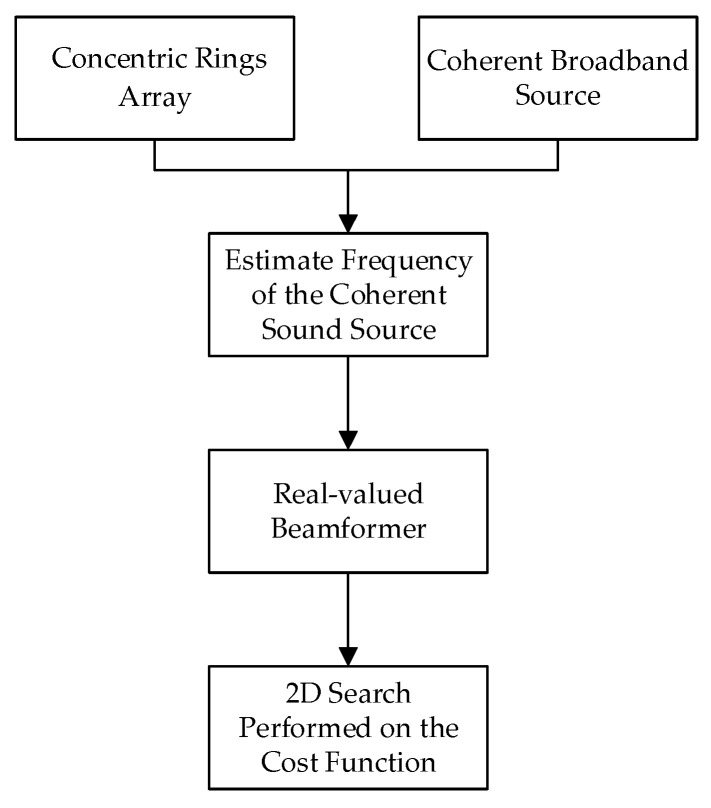
Algorithm flowchart.

Firstly, the algorithm estimates the central frequency of the coherent sound source using a frequency estimation method based on delayed data. Subsequently, it constructs a real-valued beamformer using the concept of the multi-loop phase mode. Next, it calculates the cost function within the beam space. Finally, a two-dimensional search is performed on the cost function to locate the sound source. The algorithm flow is illustrated in Figure 1. The algorithm in this article is compared with the classic MUSIC algorithm through simulation analysis in the following aspects:

(1) The relationship between Direction of Arrival (DOA) error and the center frequency of the signal source.
(2) The relationship between DOA error and the Signal-to-Noise Ratio (SNR) of the signal source.
(3) The relationship between DOA error and the number of receiving array elements.
(4) The relationship between DOA error and the array radius time.
(5) The relationship between DOA error and the circular array structure.
(6) Computational complexity.

## 2. Sound Source Model and Receiving Array Model

### 2.1. Sound Source Model

For the broadband sound source, assume that the signal bandwidth is B and that there are M mutually independent sound sources. If there are L array elements for data reception, the data received by the l array element can be expressed as:

(1) $$x_{l}(t)=\sum_{m=1}^{M}s_{m}(t-\tau_{l}{}_{m})+n_{l}(t)\;l=1,2,3\cdots L$$

where $x_{l}(t)$ represents the l-th element of the received data vector, $s_{m}(t-\tau_{l}{}_{m})$ is the sound source, and $n_{l}(t)$ is the noise data vectors. The observation time is divided into K subsegments, and then the signal source with bandwidth B is divided into J subbands. For the different frequency points $f_{1},f_{2},\cdots f_{J}$, J Equation (2) are valid. Finally, the broadband sound source model is obtained by using a discrete Fourier transform.

(2) $$X_{k}(f_{j})=A(f_{j})S_{k}(f_{j})+N_{k}(f_{j})\;k=1,2,3\cdots K;\;j=1,2,3\cdots J$$

where $X_{k}(f_{j})$, $S_{k}(f_{j})$, and $N_{k}(f_{j})$ represent the discrete Fourier transform of the received data, the discrete Fourier transform of the original sound source, and the discrete Fourier transform of the noise data vector, respectively. The size of $X_{k}(f_{j})$ is M×1, size of $A(f_{j})$ is L×M, size of $S_{k}(f_{j})$ is M×1, and size of $N_{k}(f_{j})$ is M×1.

Its array manifold $A(f_{j})$ is:

(3) $$A_{l}(f_{j})=[a_{1}(f_{j}),a_{2}(f_{j}),\cdots a_{M}(f_{j})]$$

(4) $$a_{i}(f_{j})={\left[e^{-j2\pi f_{j}\tau_{1i}},e^{-j2\pi f_{j}\tau_{2i}},\cdots ,e^{-j2\pi f_{j}\tau_{Li}}\right]}^{T}$$

The coherent sound sources differ from each other by only one complex constant. Assume that there are M coherent sound sources, namely:

(5) $$s_{m}(t)=\beta_{m}s_{0}(t)\;\;\;\;\;\;\;\;\;m=1,2,3\cdots M$$

where $s_{0}(t)$ is the broadband sound source, and $\beta_{m}$ is the complex constant. Assuming that there exists a specific relationship among the sound sources satisfying Equation (5), we combine Equation (1) to represent the measurement results of all array elements in a vector (matrix) form. By substituting Equation (5) into Equation (1), the signal model of the coherent sound source can be written as

(6) $$X(t)=A\rho s_{0}(t)+N(t)$$

where $\rho ={\left[\beta_{1},\beta_{2},\cdots \beta_{M}\right]}^{T}$ is an M×1 vector of dimensions composed of a series of complex constants, and A is the steering matrix of the matrix L×M, $N(t)=[n_{1}(t),n_{2}(t),\cdots n_{L}(t)]$.

### 2.2. Receiver Array Model

**Figure 2:**
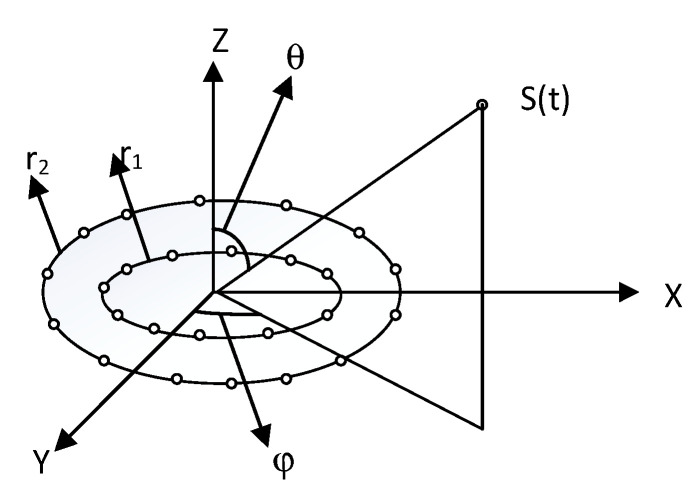
Model of a uniform circular array of two rings.

The receiving array consists of two uniform circular rings array. From the inside to the outside, the radius of the circular array is $r_{1}$ and $r_{2}$, respectively. The number of elements in the circular array inside and outside is $N_{1}$ and $N_{2}$, respectively. The array element spacing in the element space between the sensors in the same circle is $\frac{\lambda }{2}$, where λ represents the wavelength corresponding to the central frequency of the sound source. Assume that there are M sound sources, the central frequency is $f_{c}$, the bandwidth of the signal is B, and the horizontal and pitch angles of each sound source are $\theta_{i}$ and $\varphi_{i}(i=1,2,3\cdots M)$, respectively, as shown in Figure 2.

The noise received from the array is Gaussian white noise with zero mean and $\sigma^{2}$ variance. Then, the received signal of the uniform circular array can be expressed as:

(7) X(t)=AS(t)+N(t)

where $X(t)={\left[x_{1}(t),x_{2}(t),\cdots x_{N_{1}+N_{2}}(t)\right]}^{T}$ is the vector of the snapshot data. $S(t)={\left[s_{1}(t),s_{2}(t),\cdots s_{M}(t)\right]}^{T}$ is the vector of the spatial signal. $N(t)={\left[n_{1}(t),n_{2}(t),\cdots ,n_{N_{1}+N_{2}}(t)\right]}^{T}$ is the vector of noise data. A represents the steering vector array of the uniform circular array. The size of X(t) is $(N_{1}+N_{2})\times 1$, size of A is $(N_{1}+N_{2})\times M$, size of S(t) is M×1, and size of N(t) is $(N_{1}+N_{2})\times 1$.

(8) $$A=[a(f_{c},\theta_{1},\varphi_{1}),a(f_{c},\theta_{2},\varphi_{2}),\cdots a(f_{c},\theta_{M},\varphi_{M})]$$

where the steering vector:

(9) $$a(f_{c},\theta ,\varphi )=[e^{-j2\pi f_{c}\gamma_{1}},\cdots ,e^{-j2\pi f_{c}\gamma_{N_{1}}},e^{-j2\pi f_{c}\gamma_{N_{1}+1}},e^{-j2\pi f_{c}\gamma_{N_{1}+N_{2}}}]$$

where c is the speed of sound and

(10) $$\gamma_{i}=\left\{\begin{array}{c} \frac{r_{1}\cos(\theta -\frac{2\pi i}{N_{1}})\sin\varphi }{c},i=1,2,3\cdots N_{1}\\ \frac{r_{2}\cos(\theta -\frac{2\pi i}{N_{2}})\sin\varphi }{c},i=N_{1}+1,\cdots N_{1}+N_{2} \end{array}\right.$$

Since the influence of *B* on the result is not reflected in the mathematical deduction process, it can be considered theoretically that when *B* is much less than *f_c_*, that is, when the signal source is a narrow band signal, the conclusion of this paper is also applicable.

## 3. Frequency Estimation and Subspace Fitting Algorithm

The estimation of signal source frequency is one of the important ways to obtain information about the signal source. For this article, accurately estimating the center frequency of broadband signals can provide a more effective assistance for subsequent DOA results [30].

In order to ensure that the signal subspace and noise subspace in the MUSIC algorithm can be maintained as being orthogonal, the signal frequency must be estimated in advance to get the center frequency, after which the sound source can be located. In this paper, the delay data frequency estimation method is used to obtain the central frequency of the sound source. From Equation (7), we deduce that the delayed output signal vector of the array is:

(11) Y(t)=X(t−τ)=AS(t−τ)+N(t−τ)

where $\tau \leq \frac{\lambda_{\max}}{c}$, and $\lambda_{\max}$ represents the wavelength corresponding to the highest operating frequency in the entire system.

Assume that the covariance matrix corresponding to X(t) is $R_{x}$, and the cross-covariance matrix corresponding to Y(t) and X(t) is $R_{YX}$. Construct the following matrix

(12) $$R_{1}=R_{YX}R_{0}^{+}$$

where $R_{0}=R_{x}-\sigma^{2}I$. I represents the identity matrix of the same order as $R_{x}$, and + represents the pseudoinverse of the matrix. According to [6], the following relationship can be obtained:

(13) $$e^{-j2\pi f_{c}\tau_{l}}=\lambda_{l}\;\;\;\;\;\;\;l=1,2,3\cdots M$$

In Equation (13), $\lambda_{l}$ is the lth non-zero eigenvalue of $R_{1}$. Similarly, according to the different values of the delay τ in [6], there is $2\pi f_{c}\leq 2\pi \tau_{l}$, and the center frequency of the signal can be obtained according to Equation (13):

(14) $$f_{c}=-\frac{\arg(\lambda_{l})}{2\pi \tau }\;\;\;\;\;\;\;l=1,2,3\cdots M$$

where arg represents the angle of any complex number.

UCA-RB-MUSIC (Real beamforming MUSIC algorithm based on uniformly circular array) is the most common method for two-dimensional DOA estimation of the conventional uniform circular array [31,32]. The advantage of this algorithm is that a real-valued beamformer is constructed to weight the data at the receiving end of the array. And the processing of the sound source data in the beam space has the following advantages:

(1) The SNR resolution threshold and the error insensitivity are lower with the UCA-RB-MUSIC algorithm than with the UCA-MUSIC algorithm.
(2) The UCA-RB-MUSIC algorithm can use spatial smoothing technology to make sound sources decoherent.
(3) The UCA-RB-MUSIC algorithm requires only the feature decomposition of the noise subspace for a real value, which avoids the complexity of complex operations and reduces the amount of computation.
(4) The UCA-RB-MUSIC algorithm can perform parallel processing to improve operating efficiency in practical applications.

This section first introduces the concept of a phase mode; then, it constructs a real-valued beamformer to obtain the cost function of WSF in the beam space. Finally, it carries out a two-dimensional search on it to obtain the estimated value of the direction of arrival.

Let α be the polar angle of the element of the array in the polar coordinate system, ξ=2πsinφ. For a uniform circular ring array, the excitation function ω(α) is a periodic function with a period of 2π. Decompose it into a Fourier function $\omega (\alpha )=\sum_{m=-\infty }^{\infty }c_{m}e^{jm\alpha }$. For the components $c_{m}e^{jm\alpha }$, the response of the uniform circular array is $f_{m}^{c}(\theta ,\xi )=j^{{}_{m}}J_{m}(\xi )e^{jm\theta }$, where $J_{m}(\xi )$ is the Bessel function of order m. Furthermore, each response of the array corresponds to a phase pattern. For $\varphi \in [0,\frac{\pi }{2}]$, when both m>ξ and $\xi \in \left[\frac{0,2\pi r}{\lambda }\right]$ are true, $J_{m}\left(\xi \right)$ can be neglected. According to the response of the uniform circular array, the maximum number of phase modes that can be excited is $M\approx \frac{2\pi r}{\lambda }$.

For the uniform circular array of two rings proposed in this paper, the excitation sequence can be obtained by the discrete spatial sampling of the excitation function $\omega_{m}(\alpha )$ at the DOA of the uniform circular array, as follows:

(15) $$\omega_{m}^{H}=\left(\frac{1}{\left(N_{1}+N_{2}\right)}\right)[1,e^{\frac{j2\pi m}{N_{1}}},\cdots ,e^{\frac{j2\pi m(N_{1}-1)}{N_{1}}},1,e^{\frac{j2\pi m}{N_{2}}},\cdots ,e^{\frac{j2\pi m(N_{2}-1)}{N_{2}}}]$$

The response component of the array corresponding to the excitation sequence is $f_{m}^{c}(\theta ,\xi )=j^{{}_{m}}J_{m}(\xi )e^{jm\theta }+R(\theta ,\xi )$, where R(θ,ξ) is the remainder term. When the total number of elements in the array satisfies $N_{1}+N_{2}>2M$, the remainder term R(θ,ξ) can be ignored, and the response of the array can be well approximated to the continuous uniform circular array. If M is defined as the sampling frequency of space, this condition is consistent with the Nyquist sampling theorem in the time domain signal processing.

However, the algorithm in this paper needs to process broadband signals, and the value of M must be transformed along with the frequency estimation only when $M\approx \frac{2\pi r}{\lambda }$ is always satisfied, ensuring that information is not lost during the beamforming process. Thus, during the design of the array, the total number of elements must meet $N_{1}+N_{2}<\frac{2\pi r_{\max}}{\lambda_{\max}}$, where $r_{\max}$ is the radius of the outer ring.

A real-value beamformer Fr is constructed based on the phase mode satisfying the following formula:

(16) $$F_{r}^{H}=W^{H}C_{v}V^{H}$$

(17) $$W=\frac{1}{\sqrt{M}}[v(\alpha_{-m}),\cdots ,v(\alpha_{0}),\cdots ,v(\alpha_{M})]$$

(18) $$C_{v}=diag\left\{j^{-M},\cdots ,j^{-1},j^{0},j^{-1},\cdots j^{-M}\right\}$$

(19) $$v(\varphi )=[e^{-jM\varphi },\cdots ,e^{-j\varphi },1,e^{j\varphi },\cdots e^{jM\varphi }]$$

(20) $$V=\sqrt{N_{1}+N_{2}}[\omega_{-M},\cdots ,\omega_{0},\cdots \omega_{M}]$$

(21) $$M=2\pi r_{2}f_{c}/c$$

The array manifold matrix $b=F_{r}^{H}A$ of the beam space is obtained by weighting the steering vector matrix A of the array element space with $F_{r}^{H}$. $F_{r}^{H}$ is used to weight the output signals of the uniform circular array of two rings and calculates its covariance matrix. The covariance matrix is decomposed by features to obtain $R=E_{s}\Lambda_{s}E_{s}^{H}+\sigma^{2}E_{n}E_{n}^{H}$, where $\Lambda_{s}$ is composed of M maximum eigenvalues, and $E_{s}$ is a matrix consisting of the corresponding eigenvectors. $E_{n}$ is a matrix composed of eigenvectors whose eigenvalue is $\sigma^{2}$, and $\sigma^{2}$ represents the noise power.

The sound field at the position of the array elements can be expressed as $E=C\frac{e^{-jkr}}{r}S(\theta ,\varphi )$ [6,7,15,16], where C is constant. When θ and φ are the same as the actual horizontal angle and pitch angle of the sound source, the output response of the sound field is the largest. Therefore, S(θ,φ) can be regarded as a cost function. According to the output response of the real-value beamforming in (16), we try to construct a matrix or vector which makes the output response of $P_{b}E_{n}W_{opt}E_{n}^{H}$ to be the largest when θ and φ are the same as the actual horizontal angle and pitch angle of the sound source. So, the inverse matrix of $P_{b}E_{n}W_{opt}E_{n}^{H}$ can be used as a choice of S(θ,φ). There are countless cost functions in theory, and these cost functions are linearly related. The inverse matrix of $P_{b}E_{n}W_{opt}E_{n}^{H}$ is simpler. Therefore, the cost function of the two-dimensional weighted subspace fitting algorithm can be obtained.

(22) $$S_{wsf}(\theta ,\varphi )=tr(P_{b}E_{n}W_{opt}E_{n}^{H})$$

where $P_{b}=b{\left(b^{H}b\right)}^{-1}b^{H}$ is the projection matrix of the beam space steering vector matrix, $W_{opt}=(\Lambda_{s}-\sigma^{2}I)\Lambda_{s}^{-1}$ is the optimally weighted matrix, and tr(⋅) is the inverse operator of the matrix. The estimated values of the horizontal and pitch angles can be obtained using a two-dimensional search of Equation (22).

## 4. Simulation and Analysis

In order to prove the feasibility of the algorithm in this paper, the theoretical results in the above section are simulated and analyzed. Three coherent broadband sound sources are obtained by mixing different noises from the same broadband signal. The localization of the sound sources changes between [0°,90°]. The location information of the sound sources can be seen in Table 1 for details. A total of 87 positions are located respectively when frequency, SNR, number of array elements, and radius value are determined. Repeat the analysis 1000 times in each case, and take the average of 1000 times as the DOA result. At the same time, this paper uses classical MUSIC [11,12] to locate sources. Also, take the average of 1000 times MUSIC DOA results. The scanning steps of θ and φ are both 0.01 degrees. The average error of 87 positions under each simulation condition is taken as the DOA error. Assume there is no relative motion between the sound sources and the receiving array.

### 4.1. Experiments for Different Frequencies

The simulation uses a uniform circular array of two rings, with inner and outer radii of 3 m and 6 m, respectively, and the number of elements in the inner and outer ring array is 12 and 16. Keep the signal-to-noise ratio at 20 dB, change the center frequency of the sound source, and analyze the changing characteristics of the DOA error with frequency.

**Figure 3:**
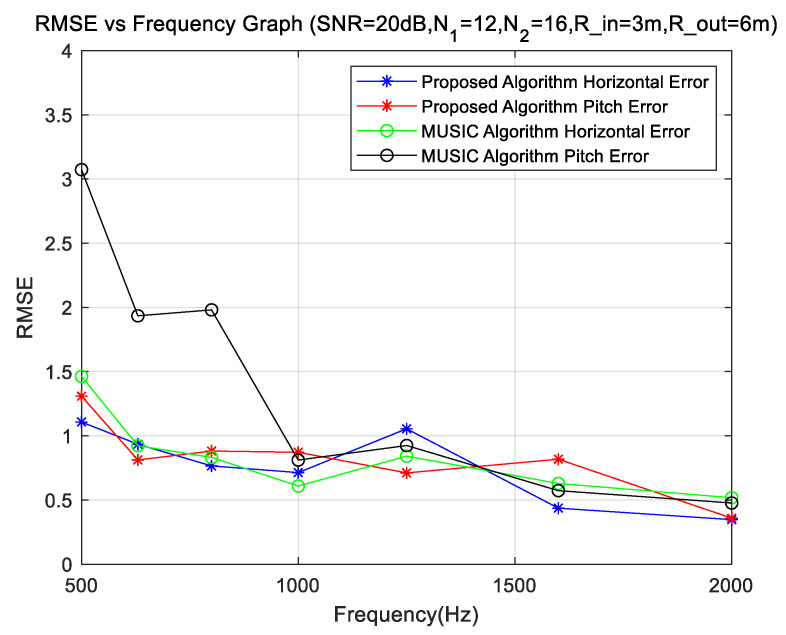
DOA error at different frequencies.

We observe that the sound sources with different center frequencies and SNR of 20 dB have different estimation effects on the estimation of DOA. Figure 3 shows the DOA error and RMES for the center frequencies of the sound sources from 500 to 2000 Hz, respectively. We observe that the horizontal angle estimation error is smaller than that of the pitch angle. The DOA method proposed in this paper is better than that of MUSIC, but the MUSIC DOA is also acceptable, and the errors are within 4 degrees. The stability of the two methods is similar, and the method proposed in this paper is slightly better. When the frequency is greater than 800 Hz, we can obtain accurate analysis results, whereas when the frequency is less than 800 Hz, we can also obtain the DOA of the sources, but the analysis results are not as good as the previous results. However, the DOA errors of the sources are significantly larger when the frequency is less than 800 Hz, which is consistent with that proposed by the equation $M\approx \frac{2\pi r}{\lambda }$.

### 4.2. Experiments for Different SNRs

The simulation uses a uniform circular array of two rings, with inner and outer radii of 3 m and 6 m, respectively, and the number of elements in the inner and outer ring array is 12 and 16. Keep the center frequency of the sound source at 1000 Hz, change the signal-to-noise ratio level from −10 dB to 25 dB, and analyze the changing characteristics of the DOA error with SNR levels.

**Figure 4:**
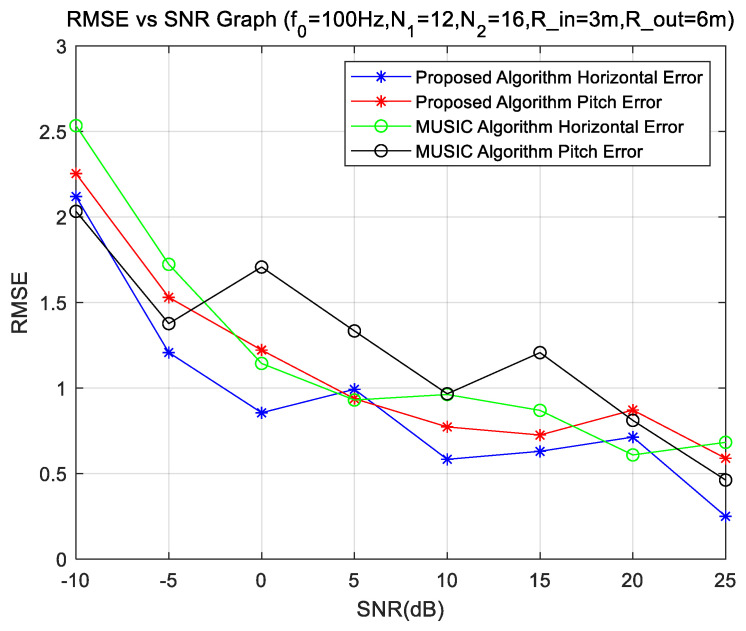
DOA error at different SNRs.

From Figure 4, we observe that at the center frequency of 1000 Hz, different SNRs have different estimation effects in the direction of arrival estimation. Figure 4 shows the DOA error of SNR between −10 dB and −25 dB. We can see in Figure 4 that the DOA results deteriorate with the reduction of SNRs, and the stability is also poor. When the SNR is greater than 5 dB, we can obtain accurate analysis results, whereas when the SNR is less than −5 dB, we can also obtain the DOA of the sources, but the DOA performance drops sharply. The method proposed in this paper has obvious advantages.

### 4.3. Experiments for Different Numbers of Sensors

The simulation uses a uniform circular array of two rings, with inner and outer radii of 3 m and 6 m, respectively. Keep the center frequency of the sources at 1000 Hz and the SNR level at 20 dB; change the number of the inner circle elements $N_{1}$ and the outer elements number $N_{2}=2N_{1}$.

**Figure 5:**
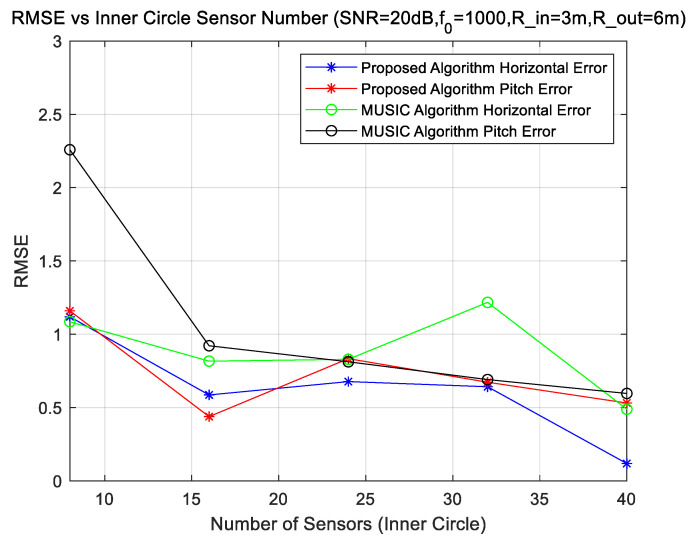
DOA error with different numbers of elements.

From Figure 5, we observe that the array element number has little effect on the DOA effect. It is not advisable to blindly increase the number of array elements in order to improve the DOA effect when the number of array elements conforms to the spatial sampling theorem.

### 4.4. Experiments for Different Radius Sizes

The simulation uses a uniform circular array of two rings, and the number of elements of the inner and outer rings array is 64 and 256, respectively. Keep the center frequency of the sound sources at 1000 Hz and SNR level at 20 dB; change the inner radii $r_{1}$ and outer radii $r_{2}=2r_{1}$.

**Figure 6:**
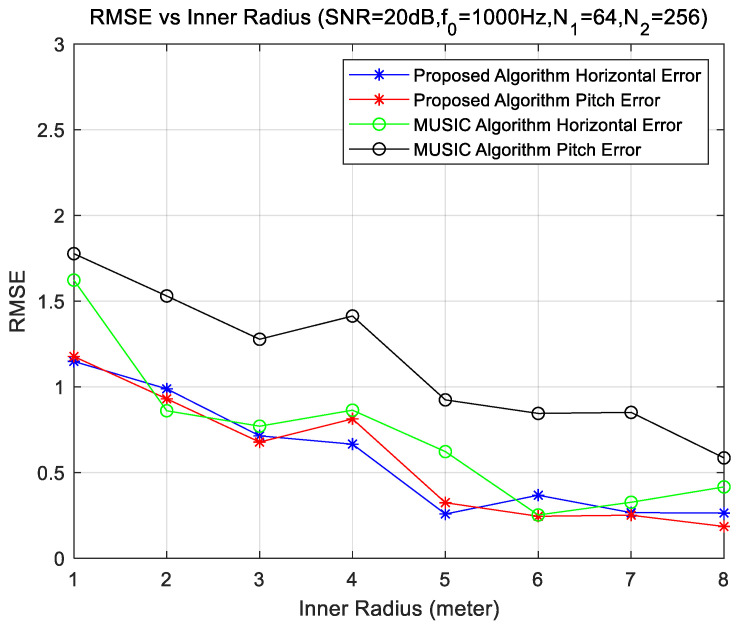
DOA error with different radii.

From Figure 6, we observe that with the increase in the radius, the DOA effect is significantly improved. Comparing Figure 4 and Figure 5, if there are no restrictions on the installation platform size of the underwater measurement array, it is advisable to first increase the radius to achieve a better DOA effect. When the radius becomes larger, the array may not conform to the spatial sampling theorem, and we can make the array conform to the sampling theorem by increasing the number of array elements. The array can be significantly improved when the simulation conditions are modified, which is consistent with the general rule $D(\theta )\propto \left(\frac{r}{c}\right)\sqrt{\frac{m}{2}}$ of the resolution of the circular array. That is, the resolution of the array increases with the increase in the array aperture and the number of array elements, where r is the radius of a uniform circular array, and m is the number of array elements.

### 4.5. Experiments for Different Structures

The simulation uses the uniform circular array of two rings vs. the uniform circular array of one ring. The numbers of array elements are equal. For a circular two-rings array, the number of inner elements is half of the outer number. Keep the center frequency of the sound sources at 1000 Hz and the SNR level at 20 dB.

**Figure 7:**
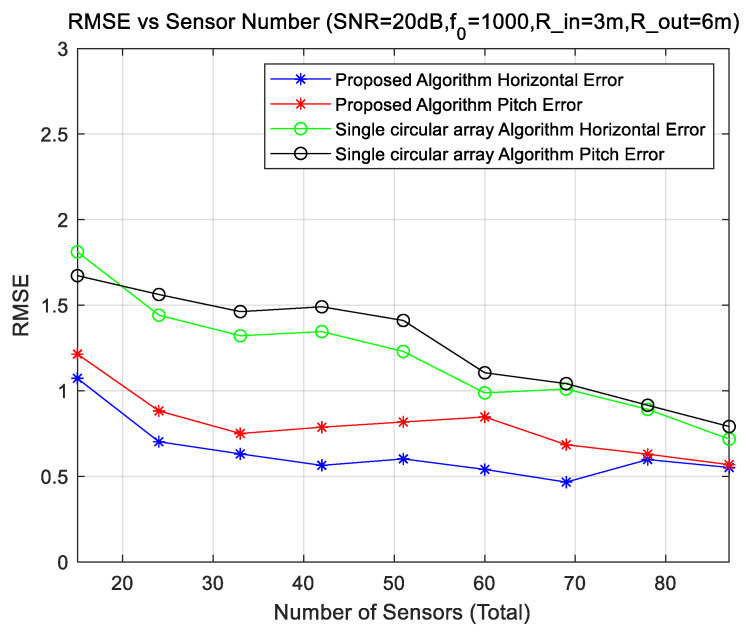
DOA error with the number of sensors.

Figure 7 shows the comparison of the positioning errors of the two formations. The figure shows the positioning error of the proposed and single circular array. Both arrays have the same aperture and the same number of sensors. It can be seen from the figure that the DOA error of the proposed method in this paper is smaller, although the DOA error of the single circular array is acceptable. When the number of sensors increases, the speed of the reduction of the DOA error becomes slow, which is consistent with the analysis results in Figure 5 and Figure 6, that is, when the number of sensors becomes large enough, the improvement of the DOA effect is very slow. The DOA error of the circular arrays for the horizontal angle of the sound source is small, and the DOA error of the pitch angle is large. Because the circular array is symmetrical with the center of the circle in the horizontal direction, the error of the horizontal angle is relatively stable, and the average error of 87 positions is naturally small. When estimating the DOA of the pitch angle, the estimation error in the 45° direction is large, resulting in the average error of the DOA estimation of the pitch angle of the circular array becoming larger. In practical applications, due to the mutual occlusion between the array elements, it is almost impossible to locate the direction of the pitch angle of 90°, which is different from the theoretical model. In the theoretical model, the 90° direction can obtain very good results because of the maximum sound path difference between the array elements. In practical applications, the estimation of sound sources DOA near a pitch angle of 90° will be almost avoided. In addition, although the DOA estimation errors of a double concentric rings array and the single circular array are close in theory, the actual DOA effect of a double concentric rings array is much better than that of the single circular array due to the occlusion between sensors.

### 4.6. Algorithm Complexity Analysis

In the preceding sections, the algorithm proposed in this paper is used to significantly improve the localization of wideband coherent signal sources compared to the MUSIC algorithm. However, the simulation and analysis make it evident that the computational time of the algorithm proposed in this paper is noticeably higher than that of the MUSIC algorithm. By comparing the computational steps of the two algorithms, it is evident that our algorithm is an optimization based on the MUSIC algorithm. It calculates the central frequency of the coherent sound sources using a frequency estimation method based on delayed data. Subsequently, it constructs a real-valued beamformer using the concept of a multi-loop phase pattern and finally computes the cost function within this beam space.

The complexity of this algorithm is noticeably higher than that of the MUSIC algorithm. To compare the complexities of the two algorithms more intuitively, this paper indirectly compares the computational complexity of the two methods by comparing their computation times. Due to the massive amount of computational data in this study, all the computations were performed on a high-performance computing server. To reduce the computation steps, both algorithms were run in the same program during actual computation, making it difficult to make a precise comparison of computation time. In order to perform a more accurate comparison of the computational complexity (runtime) of the two algorithms, this section describes the separate computation of the two algorithms on a low-performance personal computer, obtaining the analysis results of the computational complexity of the two algorithms. The program running environment is as shown in Table 2.

The computations were carried out according to the simulation conditions corresponding to Figure 3 (each data point in Figure 3 represents the statistical result after 1000 calculations), and the results are shown in Table 3:

Under the simulation conditions in the paper, due to the small scanning step, the average runtime of both methods is relatively long. Each iteration of the MUSIC algorithm requires approximately $2.7\times 10^{10}$ flops, while each iteration of the algorithm proposed in this paper requires approximately $6.8\times 10^{10}$ flops. However, according to the results in Table 3, the computational complexity of the method proposed in this article is much higher than that of the MUSIC algorithm. The computational complexity of the method in this article is approximately 2.5 times that of the MUSIC algorithm.

## 5. Conclusions

In this paper, the DOA estimation of three coherent sources is carried out using a two-ring uniform circular array. First, the center frequency is determined by using the frequency estimation method. When constructing a real-valued beamformer, the cost function is obtained, and the estimated value of the angle can be obtained by searching for this cost function. The simulation results show that the DOA method proposed in this paper has a smaller error and more stability than those of MUSIC. The angle resolution of the proposed algorithm increases with increasing number of array elements and aperture. In practical applications, increasing the number of elements in the array is a common means of improving the angle resolution. However, when the number of elements increases to a certain level, the resolution bottleneck is reached. When the number of elements of the array is unchanged, the angle resolution will be improved when only the aperture of the circular array is increased, but the spatial sampling theorem will no longer be satisfied when the array spacing is larger than half the wavelength, and the DOA performance will be sharply reduced.

## Figures and Tables

**Table 1:** Location information of sound sources.

Order Number	Source1 Localization (degree)	Source2 Localization (degree)	Source2 Localization (degree)
1	(89.55, 0.17)	(3.33, 2.33)	(89.68, 88.71)
2	(88.55, 1.17)	(4.33, 3.33)	(88.68, 87.71)
3	(87.55, 2.17)	(5.33, 4.33)	(87.68, 86.71)
4	(86.55, 3.17)	(6.33, 5.33)	(86.68, 85.71)
5	(85.55, 4.17)	(7.33, 6.33)	(85.68, 84.71)
6	(84.55, 5.17)	(8.33, 7.33)	(84.68, 83.71)
7	(83.55, 6.17)	(9.33, 8.33)	(83.68, 82.71)
…	…	…	…
83	(7.55, 82.17)	(85.33, 84.33)	(7.68, 6.71)
84	(6.55, 83.17)	(86.33, 85.33)	(6.68, 5.71)
85	(5.55, 84.17)	(87.33, 86.33)	(5.68, 4.71)
86	(4.55, 85.17)	(88.33, 87.33)	(4.68, 3.71)
87	(3.55, 86.17)	(89.33, 88.33)	(3.68, 2.71)

**Table 2:** Program running environment.

Computer Type	Personal Computer
Operating System	Windows 7 Ultimate 64-bit
Processor	Intel Core i7-6700K @ 4.00 GHz Quad-Core
Motherboard	ASUS Z170-P
Memory (RAM)	16 GB (Kingston DDR4 2400 MHz)
Primary Hard Drive	Samsung SSD 750 EVO 250 GB
Graphics Card (GPU)	Nvidia Quadro M2000 (4 GB/Dell)
Software	MATLAB 2018a

**Table 3:** Runtime of two methods.

Number of Computations	Proposed Algorithm (s)	MUSIC Algorithm (s)
100	18,496	7247
100	19,072	7188
200	42,920	17,092
200	42,075	16,968

## Data Availability

Not applicable.
